# Microbiome yarns: microbial forensics for auditing provenance in global food chains^1^^,^ 2 ^,^ 3 ^,^ 4

**DOI:** 10.1111/1751-7915.12738

**Published:** 2017-06-22

**Authors:** Kenneth Timmis, Franziska Jebok, Romilio T. Espejo

**Affiliations:** ^1^ Institute of MicrobiologyTechnical University BraunschweigBraunschweigGermany; ^2^ Institute for Educational ScienceUniversity of FreiburgFreiburgGermany; ^3^ Instituto de Nutrición y Tecnología de los Alimentos (INTA)Universidad de ChileSantiagoChile

*The Microbiome Channel, Studio 7A, BBZ Plaza, Burbank, 7.30 pm: Abigail Repor‐Tastory, Discovery Presenter, turns to face the camera*: Good evening and welcome to a new episode of ‘*Microbiome Discoveries that Change our Lives*’. Our guest this evening is once again Dr. Anastasia Noitall‐Most^5^ from the Streber Elite University of Los Angeles. Good evening Dr. Noitall‐Most *(shaking hands)* and thank you for appearing on the programme.

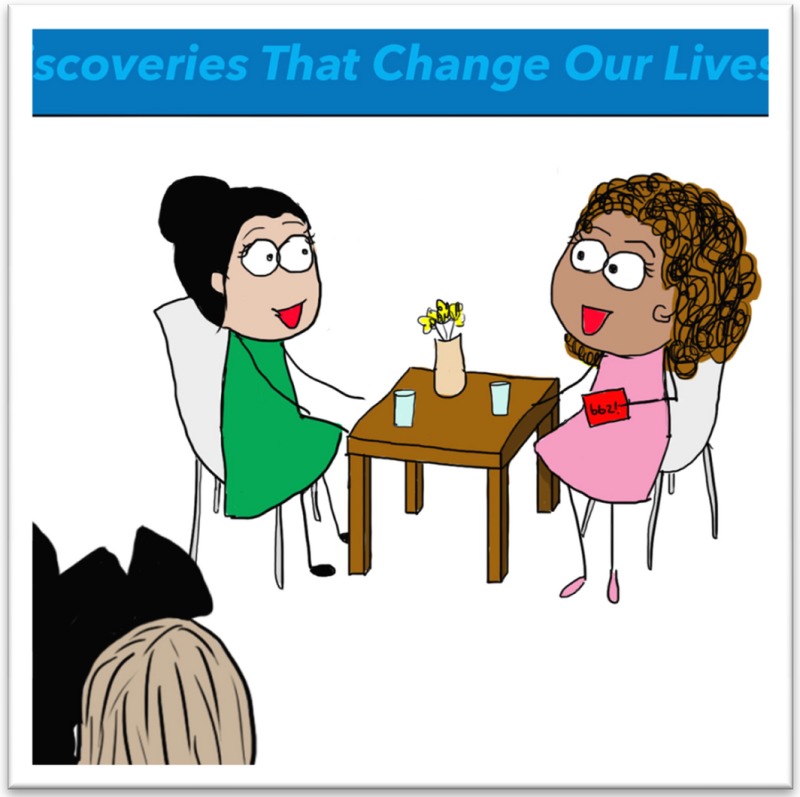

*Dr. Noitall‐Most*: Good evening Abi; it is always a pleasure to be here.

*Ms. Repor‐Tastory*: Ani, this evening we want to discuss a new development that addresses a very scary contemporary problem we are all faced with, namely food safety in the global economy.

*Dr. Noitall‐Most*: Yes, Abi, this is a very reassuring advance by the group of Gill Jackbert at the Gulf Fisheries Microbiome Centre at UT – Houston, and Forensics4FoodProvenance Inc., a company he created in Tampa.

But first a bit of background. As you know, with globalization, the food that ends up on our table, or on the restaurant table, can have been produced practically anywhere in the world. While this has major positive consequences, for example we can have the health benefits of fresh broccoli all the year round, it brings with it security challenges because national safety practices and regulations are different in different countries.

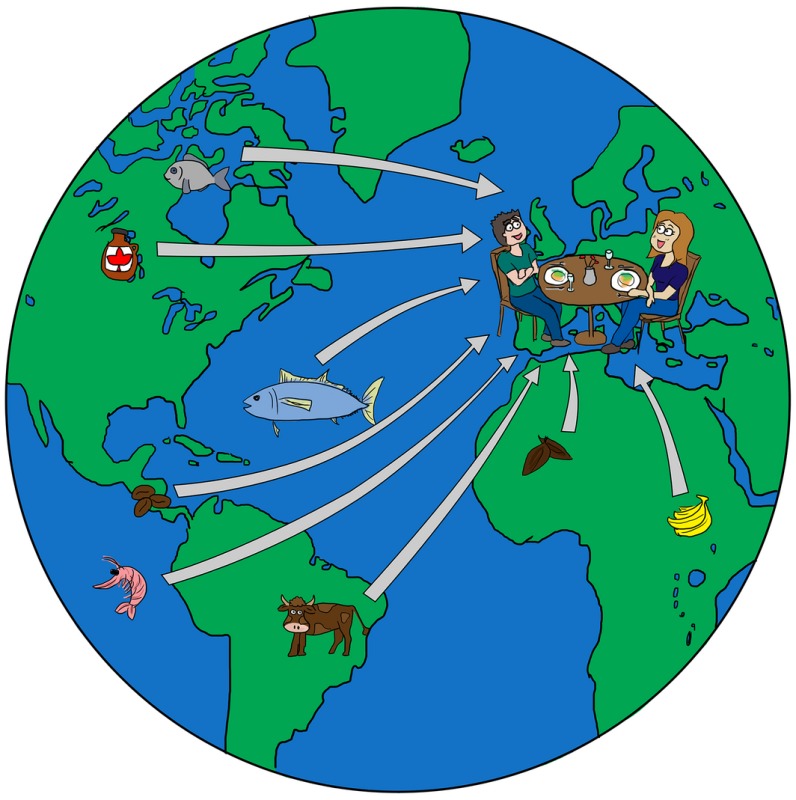

This is especially true with regard to hazards like contamination with toxic chemicals and biochemicals, and infectious agents. Of course, our authorities do their utmost to assure food safety by elaborating regulations for imported products, but the odd dodgy importer can be quite creative in circumventing these. The alternative is of course to check products as they come into the country, but, until now, this has represented a massive, essentially impossible task. So, we are obliged to rely largely on provenance and safety documentation provided by the exporter/importer, and/or on local supervision by agents from the importing country. Unfortunately, although most exporters/importers rigorously adhere to our domestic safety standards, there are a few black sheep who are quite inventive in obfuscating provenance, or in exploiting legal re‐exporting possibilities that circumvent restrictions.^7^

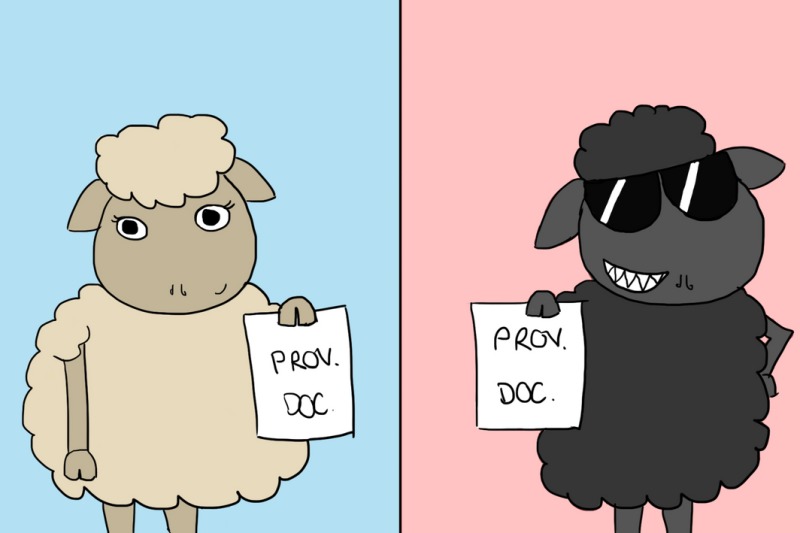

*Ms. Repor‐Tastory*: Okay, Ani: so what kind of problems are we talking about?

*Dr. Noitall‐Most*: Well, one product well known to be associated with quite a bit of hazard is seafood, which may contain pathogens, like vibrios,^8^ that produce diseases ranging from diarrhoea to systemic lethal infection, and algae, some of which produce potent toxins, including the red tide lethal neurotoxin.^9^ One reason for this is that molluscs like clams and oysters are so‐called filter feeders, which means they nourish themselves by filtering the water they live in for food particles, including microbes. Some microbes filtered out are, however, not digested but survive in the animal. In fact, some pathogens, like us, very much like seafood and actually colonize and multiply in the clam, mussel, shrimp, etc., and reach us via this route. Toxic algae also filter‐accumulate in seafood this way. So even with best practice hygiene measures of handling, our little shellfish friends on the table can bite back.

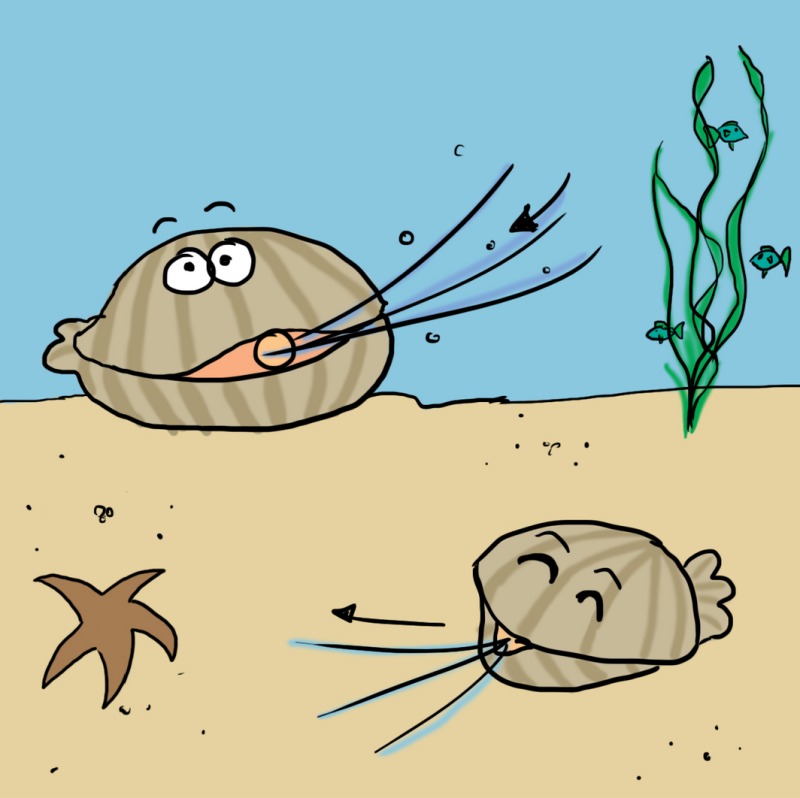

Moreover, seafood produced in aquaculture facilities may additionally have rather high levels of toxic metals/metalloids, like cadmium and arsenic, or be contaminated by human or animal waste that can contain other microbial and viral pathogens. And while cooking destroys most pathogens, it also changes the flavour, so some people prefer to eat seafood raw and thereby swallow the full dose.

Of course, even cooking is not a sure‐fire means of eliminating microbes and their products from food – for example the lethal neurotoxin produced by algae causing red tides survives cooking^9^ – and poor hygiene in the handling of the product before and after cooking provides the opportunity for transmission of pathogens directly to people or indirectly via other foods in the kitchen. All of this can lead to some not‐so‐pleasant toilet experiences – assuming of course the toilet can be reached in time.

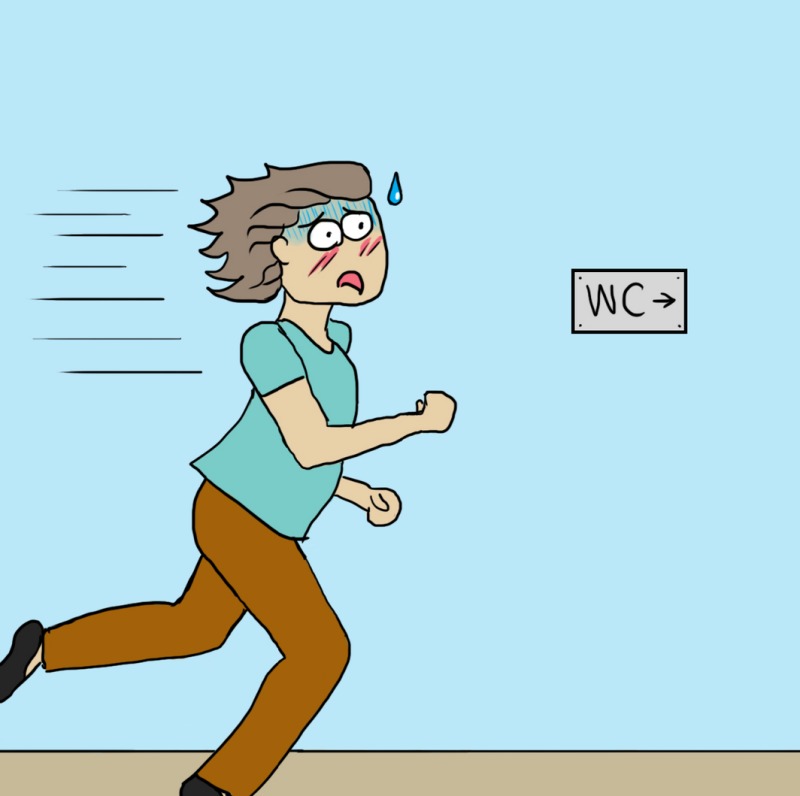

*Ms. Repor‐Tastory, looking concerned, her right hand migrating involuntarily to her mouth, her left hand descending to her abdomen*: Yes, well, having had a shrimp‐mayo wrap for lunch, I imagine this can be an unpleasant surprise.

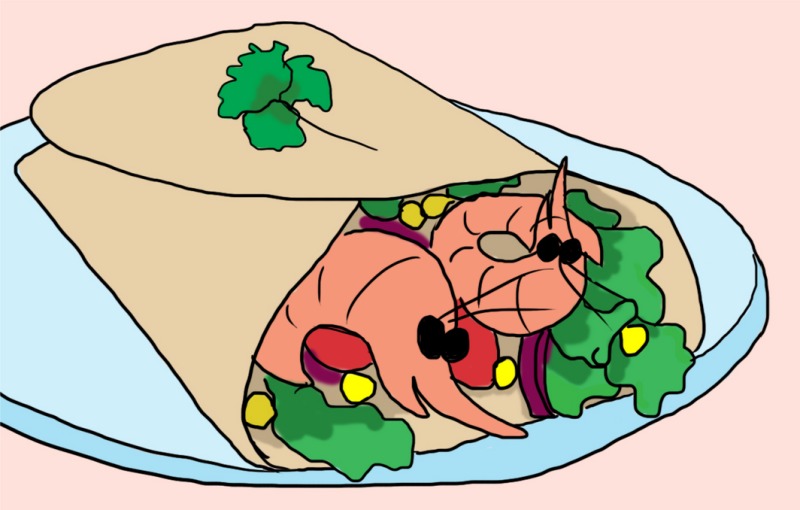

*Dr. Noitall‐Most*: Yes, I know what you mean. A number of years ago, I was invited by a famous toxinologist friend from the Pasteur Institute in Paris for a plateau de fruits de mer in Montparnasse, a plateau containing both cooked and uncooked seafood.

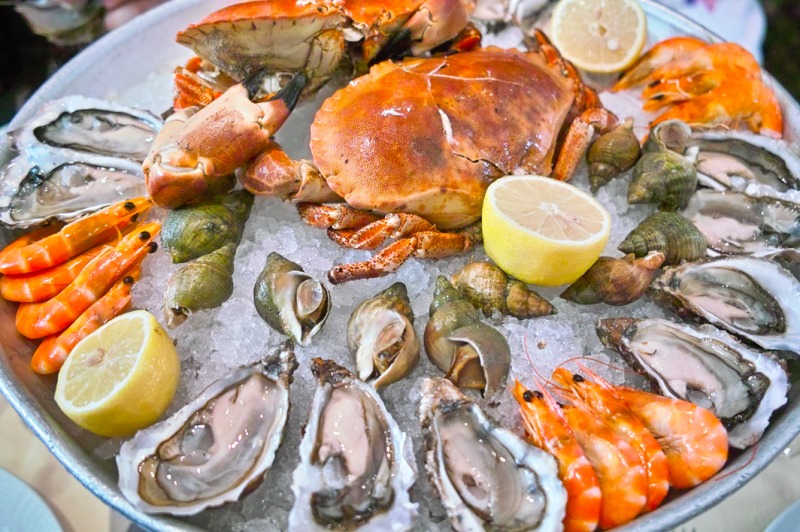

Image under CreativeCommons 2.0 Licence, source: https://www.flickr.com/photos/13523064@N03/3764045059

The evening was truly memorable, not only because of the excellent company, the delicious food and very tolerable Sancerre, but also because, in the evening return TGV, almost at my destination, I experienced the most explosive…

*Ms. Repor‐Tastory, audible noises emanating from the in‐ear headphone in her left ear and making frantic facial warning signs…*

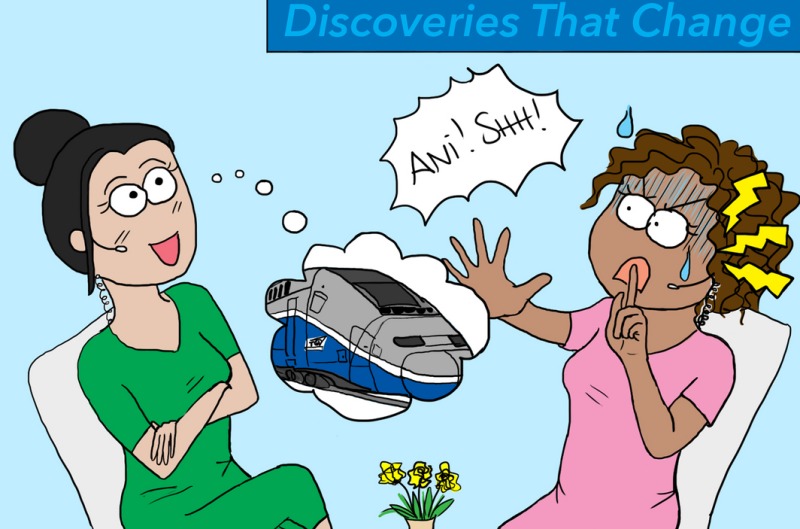

*Dr. Noitall‐Most*: Oh, sorry, I am getting carried away! Where was I? Oh yes; but this is perhaps not the worst aspect, because you as an individual sitting glum on the toilet may be unfortunate, but do not represent a wider problem to the world. More scary is the problem of antibiotic resistance which is considered by some to be the number one global health challenge of our time.^10^ The appearance of superbugs resistant to drugs of last resort means that it is becoming impossible to treat some infections.

*Ms. Repor‐Tastory*: Okay. Yes, Ani, there has been a lot of this stuff in the press recently, but what does this have to do with my shrimp‐mayo wrap and microbiome science?

*Dr. Noitall‐Most*: Actually everything, Ali. Previously, antibiotic resistance was a natural feature of a few bugs, but not of most microbes in the environment, and fortunately not most pathogens causing infections. Antibiotic sensitive bugs are generally fitter than resistant ones, so beat the resistant ones down. This is why clinicians have been able to treat infections with antibiotics so successfully for so long. Unfortunately, antibiotics became overprescribed, providing a powerful selection pressure for the development and spread of antibiotic resistance among microbes. Even more important, the finding that antibiotics also have prophylactic benefits in animal husbandry led to their massive global use in food animal rearing, including aquaculture.^11^ This practice has now contaminated the planet with a class of highly active compounds that inexorably convert our wonderful antibiotic sensitive friends, which carry out so many diverse tasks important to humankind, to drug resistance and from which our microbial enemies – the pathogens – acquire their resistance. As a result, difficult‐to‐treat infectious diseases have returned with a vengeance and the proportion of non‐curable infections is growing inexorably.

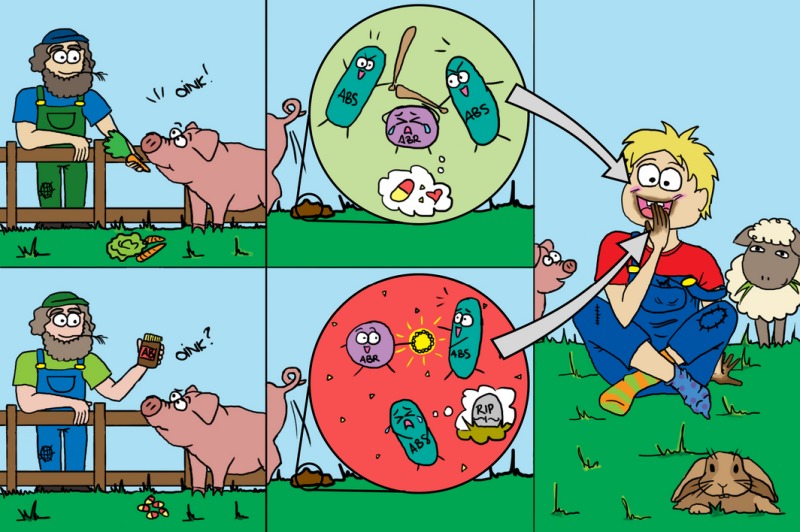

So, the connection with your shrimp‐mayo wrap is as follows: shrimp harvested from pristine waters containing little or no antibiotics may contain a few pathogens and/or trace amounts of algal toxins, but are not generally hazardous. If contaminating pathogens cause an infection, they are largely drug sensitive and hence easily treatable. However, shrimp from some aquaculture operations that use antibiotics as growth promoters may contain pathogens, some of which may be drug resistant and hence difficult to treat.

*Ms. Repor‐Tastory*: So how can we know whether the shrimp comes from pristine or contaminated environments? Is the fishmonger‐supermarket‐restaurant able to tell us?

*Dr. Noitall‐Most*: This is of course the crux of the problem and the topic of the discovery we discuss this evening. As I mentioned earlier, the provenance of some items of food traded globally is problematic, and this is particularly true of the products of aquaculture, as exemplified by shrimp. Obviously, documentation can be helpful but reliance on documentation does not give adequate consumer protection. So, there needs to be documentation‐independent provenance determination during the process of importation. Previously, there were no adequate procedures for determining the provenance of seafood, but now, Dr. Jackbert and his colleagues seem to be on the way to solving the problem.

I met Gilly recently at a meeting on food safety in Dublin and he told me that the microbial flora of the digestive tract of the shrimp – you know: the black vein you see when you remove the shell, also called the poo thread, which some people remove but others consider to be an essential component of the authentic flavour experience – constitutes a signature that is unique to each aquaculture operation. While determining the complete microbial signature of a shrimp is a bit time‐consuming and expensive, Gilly's group has identified certain key microbes responsible for most of the signature uniqueness and have used this to develop a rapid, relatively inexpensive screening procedure.

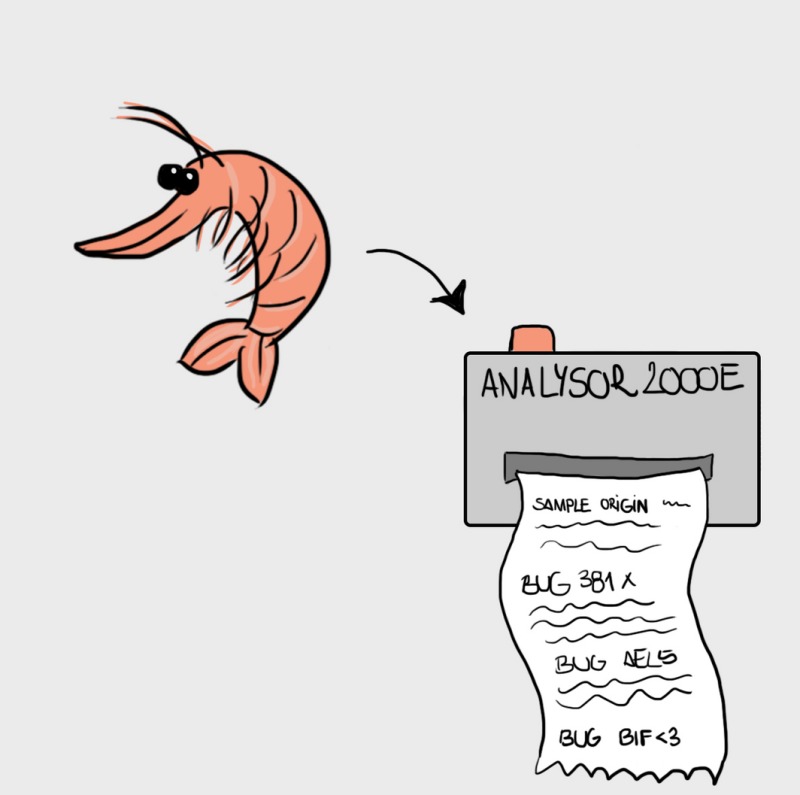

*Ms. Repor‐Tastory*: Well that is wonderful! So how many different microbes make up this shrimp signature?

*Dr. Noitall‐Most*: Ali, we do not have detailed information about the test at the moment. I understand that it is currently being patented and until the patent is awarded and published, key details will remain intellectual property of F4FP.

But of course, the really exciting thing is that this approach is simply the harbinger of a revolution. The race is on to determine microbial provenance signatures for all manner of globally traded foods, which will represent an exquisitely sensitive new type of food forensics, similar to the recently developed human microbiome forensics,^12^ that can be applied at all parts of the supply chain. If this approach fulfils its promise, we will gain a much higher level of food safety in a world of global supply.

*Ms. Repor‐Tastory*: Wow, that vision is really exciting! And it means that I will be able to enjoy my shrimp‐mayo wraps with impunity!

*Dr. Noitall‐Most*: Of course, Ali, at least in terms of treatability of any infection with explosive consequences it may gift you.

++++++++++++++++++++++++

## Explanatory notes for online version at www.theabsurdmicrobe.com

## Notes

5 youngest daughter of the renowned Dr. Noitall, see e.g. Koshland, D. E., Jr. 1994. Editorial: A simple proposal for health care rationing. Science **264**: 1515.

7 e.g. see https://www.bloomberg.com/news/features/2016-12-15/how-antibiotic-tainted-seafood-from-china-ends-up-on-your-table; http://www.thetimes.co.uk/article/chinese-ban-on-crabmeat-costs-millions-krcj73thk.

8 e.g. see Colwell, R. R. and Liston, J. 1960. Microbiology of shellfish. Bacteriological study of the natural flora of Pacific oysters (*Crassostrea gigas*). Appl. Microbiol. 8: 104–109; Krantz, G. E., Colwell, R. R. and Lovelace, E. 1969. *Vibrio parahaemolyticus* from the blue crab *Callinectes sapidus* in Chesapeake Bay. Science 164: 1286–1287; Huq, A., Small, E. B., West, P. A., Huq, M. I., Rahman, R. and Colwell, R. R. 1983. Ecological relationships between *Vibrio cholerae* and planktonic crustacean copepods. Appl. Environm. Microbiol. 45: 275‐ 283; Fuenzalida, L., Hernandez, C., Toro, J., Rioseco, M. L., Romero, J. and Espejo, R. T. 2006. *Vibrio parahaemolyticus* in shellfish and clinical samples during two large epidemics of diarrhoea in southern Chile. Environm. Microbiol. 8: 675–683; Jones, M. K. and Oliver, J. D. 2009. *Vibrio vulnificus*: disease and pathogenesis. Infect. Immun. 77: 1723–1733; Garcia, K., Bastias, R., Higuera, G., Torres, R., Mellado, A, Uribe, P. and Espejo, R. T. 2013. Rise and fall of pandemic *Vibrio parahaemolyticus* serotype O3:K6 in southern Chile. Environm. Microbiol. 15: 527–534.

9 Van Dolah, F. M. 2000. Marine algal toxins: origins, health effects, and their increased occurrence. Environm. Health Persp. 108: 133–141; Au.: detection of red tide‐associated neurotoxin in mollusc‐rearing regions leads to immediate discontinuation of harvesting, to prevent neurotoxin transfer to customers.

10 World Health Organization. 2015. Global action plan on antimicrobial resistance.

11 e.g. see Levy, S. B. 1971. The problems of drug‐resistant pathogenic bacteria. Studies on R factors segregated into E. coli minicells. Ann. N. Y. Acad. Sci. 182: 217–225; Levy, S. B., FitzGerald, G. B. and Macone, A. B. 1976. Spread of antibiotic‐resistant plasmids from chicken to chicken and from chicken to man. Nature 260: 40–42; Levy, S. B. 1982. Microbial resistance to antibiotics. An evolving and persistent problem. Lancet 8289: 83–88; Cabello, F. C. 2006. Heavy use of prophylactic antibiotics in aquaculture: a growing problem for human and animal health and for the environment. Environm. Microbiol. 8: 1137–1144; Cabello, F. C., Godfrey, H.P., Buschmann, A. H. and Dölz, H. J. 2016. Aquaculture as yet another environmental gateway to the development and globalisation of antimicrobial resistance. Lancet Infect Dis. 16: e127–33. https://doi.org/10.1016/s1473-3099(16)00100-6; Cabello, F. C. and Godfrey, H. P. 2017. Comment on: Transferable resistance to colistin: a new but old threat. J. Antimicrob. Chemother. 72: 636–637.

12 Hampton‐Marcell, J. T., Lopez, J. V. and Gilbert, J. A. 2017. The human microbiome: an emerging tool in forensics. Microb. Biotechnol. 10: 228–230.

